# Metabolic Maturation of Auditory Neurones in the Superior Olivary Complex

**DOI:** 10.1371/journal.pone.0067351

**Published:** 2013-06-27

**Authors:** Barbara Trattner, Céline Marie Gravot, Benedikt Grothe, Lars Kunz

**Affiliations:** 1 Department of Biology II, Division of Neurobiology, Ludwig Maximilians University Munich, Martinsried, Germany; 2 Graduate School of Systemic Neurosciences, Ludwig Maximilians University Munich, Martinsried, Germany; McGill University, Canada

## Abstract

Neuronal activity is energetically costly, but despite its importance, energy production and consumption have been studied in only a few neurone types. Neuroenergetics is of special importance in auditory brainstem nuclei, where neurones exhibit various biophysical adaptations for extraordinary temporal precision and show particularly high firing rates. We have studied the development of energy metabolism in three principal nuclei of the superior olivary complex (SOC) involved in precise binaural processing in the Mongolian gerbil (*Meriones unguiculatus*). We used immunohistochemistry to quantify metabolic markers for energy consumption (Na^+^/K^+^-ATPase) and production (mitochondria, cytochrome c oxidase activity and glucose transporter 3 (GLUT3)). In addition, we calculated neuronal ATP consumption for different postnatal ages (P0–90) based upon published electrophysiological and morphological data. Our calculations relate neuronal processes to the regeneration of Na^+^ gradients perturbed by neuronal firing, and thus to ATP consumption by Na^+^/K^+^-ATPase. The developmental changes of calculated energy consumption closely resemble those of metabolic markers. Both increase before and after hearing onset occurring at P12–13 and reach a plateau thereafter. The increase in Na^+^/K^+^-ATPase and mitochondria precedes the rise in GLUT3 levels and is already substantial before hearing onset, whilst GLUT3 levels are scarcely detectable at this age. Based on these findings we assume that auditory inputs crucially contribute to metabolic maturation. In one nucleus, the medial nucleus of the trapezoid body (MNTB), the initial rise in marker levels and calculated ATP consumption occurs distinctly earlier than in the other nuclei investigated, and is almost completed by hearing onset. Our study shows that the mathematical model used is applicable to brainstem neurones. Energy consumption varies markedly between SOC nuclei with their different neuronal properties. Especially for the medial superior olive (MSO), we propose that temporally precise input integration is energetically more costly than the high firing frequencies typical for all SOC nuclei.

## Introduction

In spite of the importance of energetics for neuronal function, very few studies have addressed the relationship between neuronal energy supply and metabolic demands [1], [2], [3]. Quantitative studies, which have been published so far, focussed mainly on the cerebellum, the cerebral cortex and the olfactory glomerulus [4], [5], [6], [7], but neurones in brainstem nuclei have not yet been investigated in this context. The neurones of the superior olivary complex (SOC) in the mammalian auditory brainstem, which are responsible for localising sounds in space, exhibit various biophysical adaptations (e.g. a very low input resistance) that facilitate fast and temporally accurate auditory processing and display some of the highest firing rates in the brain. These neurones are, therefore, of special interest from the standpoint of neuroenergetics, as also measurements by Sokoloff and colleagues revealed high rates of glucose utilisation in auditory nuclei (for a review see [8]).

We have monitored developmental changes in the SOC of the Mongolian gerbil (*Meriones unguiculatus*), a model organism whose auditory system shows broad similarity to that of humans with respect to its frequency-response profile and the organisation of its brainstem [9], [10], [11]. More specifically, we have investigated the three SOC nuclei involved in precise binaural processing, i.e. the medial superior olive (MSO), the lateral superior olive (LSO) and the medial nucleus of the trapezoid body (MNTB). Notably, the calyx of Held synapse in the MNTB allows for extremely fast, efficient and reliable synaptic information transfer, resulting in firing rates of up to several hundred hertz, during phase-locking to the fine-structure or the envelope of acoustic stimuli for instance [12], [13]. These properties are pivotal prerequisites for the function of the MNTB in computing the location of sound sources in space, thereby enabling orientation and navigation using auditory cues [14], [15], [16], [17], [18], [19], [20]. Amplitude differences between the sounds reaching the two ears − so-called interaural level differences − are encoded in the LSO [21], [22], [23], while differences in arrival times − so-called interaural time differences − are extracted by the MSO [14], [18], [24].

Developmental changes in energy consumption have only rarely been at the focus of neuroenergetics studies [25]. Our aim here was to investigate how the metabolic activity of SOC neurones changes during early postnatal stages, a period during which these neurones show the profound functional alterations that underpin the evolving ability to hear. In gerbils this ability first appears on postnatal day 12–13, as the rodents gradually become sensitive to auditory stimuli [26]. The developmental regulation of energy availability in the auditory brainstem is interesting, because neuronal circuits are already fully formed prior to hearing onset, but are driven only by spontaneous activity from the cochlea [27], [28], [29], [30], [31], [32], [33]. Furthermore, it is known that this “precocious” and often synchronous burst firing helps to refine the tonotopic organisation of the SOC nuclei [34], [35]. After hearing onset, SOC neurones undergo an additional physiological maturation (for review on the LSO see [36]) during which biophysical properties [37], [38], [39], [40], [41], [42], cell morphology [31], [43], [44], [45], neuronal connectivity [31], [46], [47] and response patterns [48], [49] are fine-tuned in accordance with the auditory demands. In order to achieve the precision and speed required for accurate auditory perception, these neurones obviously have to develop the metabolic capacity to sustain this capability.

To address the issue of neuroenergetics and the development of its constituent processes in the auditory brainstem, we employed two complementary approaches using a mathematical model to calculate energy consumption during neuronal activity and immunohistochemical methods to quantify levels of selected metabolic markers in MSO, LSO and MNTB. We calculated the energy consumption of these neurones during development based on electrophysiological and morphological parameters for animals of different ages taken from the literature. In many nuclei of the auditory system, development before and after hearing onset coincides with changes in the electrophysiological and morphological properties (for references see above). Our mathematical modelling is based on the approach used by Attwell and others, which relates all neuronal processes to the regeneration of the perturbed Na^+^ gradient and consequently to the energy required to power the membrane Na^+^/K^+^-ATPase [50], [51], [52], [53]. The energy-consuming processes we considered were the maintenance of resting membrane potential, the generation of action potentials and postsynaptic excitatory currents, since these have been shown to be the most prominent energy users in other neurones and are closely related to the location of the metabolic markers we have quantified.

We analysed and compared expression levels of several metabolic markers in the three SOC nuclei. As cytochrome c oxidase (COX) catalyses a crucial step in mitochondrial adenosine-5′-triphosphate (ATP) production, its activity can thus serve as a direct read-out of mitochondrial efficiency and neuronal activity [51], [52]. In both the inferior colliculus and in the endbulb of Held synapse in the cochlear nucleus, a developmentally regulated increase in mitochondrial density has already been shown to coincide with hearing onset [53], [54]. The Na^+^/K^+^-ATPase plays a fundamental part in neural excitation and firing since it maintains an asymmetric ion distribution across the cell membrane and restores ionic gradients during neuronal activity [55]. Thus, Na^+^/K^+^-ATPase activity initiated by neuronal activity is tightly coupled to mitochondrial ATP production [55], [56]. Glucose, as the main metabolic substrate for ATP production in the mammalian brain is transported into neurones by glucose transporters (GLUTs), with GLUT3 being the predominant subtype [57], [58], [59], [60]. Indeed, the up-regulation of GLUT3 appears to be tightly correlated with functional activity and neurotransmission [58], [60], [61], [62].

## Materials and Methods

### Ethics Statement

The experiments described in the following were in compliance with institutional guidelines, and with State (Bavarian) and German Federal laws, and were carried out in accordance with the European Communities Council Directive of 24 November 1986 (86/609/EEC). The local government of Upper Bavaria (Regierung von Oberbayern) approved the study (Ref. No. 55.2-1-54-2531-105-10).

### Animals

We used 21 animals altogether, of both sexes, for this study: 12 for immunohistochemical stainings (2 in each age group), and 9 for COX activity stainings (3 animals at P7; 2 at P10, P14, and P30, respectively). Animals in the same age group were from different litters. For both types of experiments, animals were anaesthetised using 100 mg/kg body weight metamizol (Novalgin®, sanofi aventis) p.o., followed by 200 mg/kg body weight pentobarbital (Narcoren®, Merial GmbH, Halbergmoos, Germany) i.p. After the animals had reached a state of deep anaesthetic stage, marked by a complete loss of the flexor reflex at all limbs, they were perfused with Ringer solution supplemented with 0.1% heparin (Meditech Vertriebs GmbH, Parchim, Germany) at a flow rate of 4 ml/min for 10 min followed by 4% paraformaldehyde (PFA) solution for 20 min. Brains were then post-fixed overnight in 4% PFA at 4°C.

### Immunohistochemistry

Using a Leica VT1200S vibratome, 50 µm sections of the auditory brainstem were collected. The sections were washed 4 times in 0.1 M PBS for 5 min each. Non-specific binding sites were saturated with a blocking solution containing 1% BSA and additionally 0.3% Triton X-100 and 0.1% saponin to allow better penetration of the antibodies into the tissue. The sections were incubated in this blocking solution for 1 hour at room temperature on a shaker. The sections were then incubated in the primary antibody mix (diluted in blocking solution) overnight at 4°C on a shaker. The specificity of all primary antibodies used has been previously published and the relevant publications are indicated for the respective antibodies. The primary antibodies used were: chicken anti-Map2 (1∶1000, Neuromics, CH22103 [45], [63]), mouse anti-ATPase (1∶1000, DSHB, a5 [64], [65]), rabbit anti-synapsin (1∶100, SySy, 106 002 [66]), rabbit anti-GLUT3 (1∶100, abcam, ab41525 [62], [67]) and mouse anti-mitochondria (1∶500, abcam, ab3298 [68]). The anti-mitochondria antibody was raised against a non-glycosylated protein component of the mitochondrial membrane obtained from a partially purified mitochondrial preparation. The anti-GLUT3 antibody is directed against the intracellular C-terminal of GLUT3. Therefore intracellular as well as membrane-bound GLUT3 is labelled [62], and the intracellular GLUT3 fraction is detectable as a consequence of the relatively fast turnover rate of the protein [69]. Every staining always contained an anti-Map2 co-staining to enable the determination of the cell size, which was important during our subsequent analysis. In addition, stainings for Na^+^/K^+^-ATPase were always carried out together with anti-synapsin staining. Next day, sections were washed 4 times in 0.1 M PBS for 5 min each, and then incubated with the appropriate secondary antibodies for 2–3 hours at room temperature on a shaker. Secondary antibodies used were: donkey anti-chick Cy3 (1∶300, Dianova, 703-166-155), goat anti-rabbit Alexa488 (1∶400, Molecular Probes, A-11034) and goat anti-mouse DyLight 649 (1∶300, Dianova, 115-495-205). Finally, the tissue slices were mounted with Vectashield supplemented with DAPI (H-1200, Vector). We have standardised our immunohistochemical stainings by using the protocol described above to ensure equal conditions for all sections. In addition, we ensured that no staining gradient (e.g. due to overlaying sections or adhering of the section to the staining tank) was present in those sections used for analysis.

### COX Activity

For analysis of COX activity [51], [70], animals were perfused and brains were treated as described above, except that post-fixation in 4% PFA was terminated after only 4 hours to avoid denaturation of the enzyme. Sections of the auditory brainstem were prepared as outlined above, washed 4 times in 0.1 M PBS and subsequently incubated in staining solution (containing 60 mg 3,3′-diaminobenzidine (Sigma Aldrich, D5637) and 20 mg cytochrome *c* (Sigma Aldrich, 30398)/90 ml 0.1 M PBS) in the dark at 37°C until the colour reaction developed. We made sure to incubate all the slices that needed to be directly compared in the same staining solution for the same time. Hence, the sections from the different age groups in each experiment were always incubated together in the same solution for the same period of time. This ensured that no intensity differences due to variations in the reactivity of the enzyme appeared between age groups. Note that, however, the intensity of COX staining might be varying between different sets of experiments. Sections were washed 3 times in 0.1 M PBS, mounted on gelatine-coated slides, subsequently dehydrated and embedded in malinol.

### Image Acquisition

To visualise immunohistochemical stainings, confocal optical sections were acquired with a Leica 6000CS SP5 confocal laser-scanning microscope (Leica Microsystems, Mannheim) equipped with a Plan 63×/NA1.32 oil immersion objective. Fluorochromes were visualised by using an argon laser with an excitation wavelength of 488 nm (emission 494–555 nm for Alexa488), a DPSS laser with a laser line at 561 nm (emission 565–606 nm for Cy3) and a helium-neon laser with an excitation wavelength of 633 nm (emission 640–740 nm for DyLight649). For each optical section the images were collected sequentially for the different fluorochromes. Stacks were obtained with axial distances of 300 nm – the image size was 512×512 pixels. The voxel size was either 60.18 nm×60.18 nm×300 nm (zoom 8×) or 160 nm×160 nm×300 nm (zoom 3×). To improve the signal-to-noise ratio, each section image was averaged from six successive line scans. The cells analysed in this study were selected at random and originated from different sections. Acquisition and analysis of images was performed by observers, who were unaware of the age of the animal.

### Image Editing and Quantification

After stack acquisition, we corrected Z chromatic shift between colour channels. RGB stacks, montages of RGB optical sections and maximum-intensity projections were generated by using *ImageJ* (1.39q Wayan Rasband, National Institutes of Health, USA, http://rsb.info.nih.gov/ij/, Java 1.5.0_06) and *Adobe Photoshop* (8.0.1) software. Quantification of the stainings was performed by a custom-written thresholding procedure in *ImageJ*. We obtained the cross-sectional area of the neurone by drawing the contour within a single optic plane using the Map2 staining pattern as an indicator, as the expression level of this marker remains constant during the different developmental stages in the soma of the cells. The threshold square was positioned in an area where no specific staining of the respective antigen was observed (nucleus). The site and area of the nucleus was identified by DAPI staining. For the determination of the threshold a scaling factor of 5 was employed, as this factor showed the best magnification level for the images. The program then computed the number of pixels within the cell area that lie above the threshold, which was determined beforehand. The percentage of the pixels above the threshold gave us the percentage of the soma, which was positive for the antigen of interest (mitochondria, Na^+^/K^+^-ATPase or GLUT3) and represents the marker level (in % per area). To avoid a bias of the plane of the confocal image within the cell, we averaged across several optical sections within a single cell. To rule out any bias in the data due to a non-uniform distribution of staining across the cell, we checked the distribution of the respective antigen in some sample cells across all optical sections (n = 5 cells) and verified that the percentage of positive pixels was highly uniform (data not shown).

Statistical analysis of the immunohistochemical quantifications for the different age groups was performed using the software *Prism5* (5.00 for Windows, GraphPad Software, San Diego California USA, www.graphpad.com). Statistical dependence between metabolic marker levels and age was analysed by means of the nonparametric Spearman correlation test. The parameters obtained (Spearman correlation coefficients; P values) are given in the figure legend.

### Energy Calculations

All calculations of energy consumption by different processes are based on the assumption that regeneration of resting Na^+^ and K^+^ gradients by the Na^+^/K^+^-ATPase consumes one ATP molecule for pumping two K^+^ ions in and three Na^+^ ions out [71], [72], [73], [74]. Therefore, all energy values are expressed as number of ATP molecules consumed per second and per cell. The relevant energy-utilising processes considered were (1) maintenance of resting membrane potential, (2) generation of action potentials in soma and dendrites and (3) postsynaptic AMPA currents. E_Vr_, the energy required for maintenance of resting membrane potential (V_r_) is assumed to be determined by the Na^+^ current (I_Na_) driven by the Na^+^/K^+^-ATPase, which maintains the Na^+^ (and K^+^) gradient across the plasma membrane. Thus, E_Vr_ depends on V_r_, input resistance (R_in_) and the reversal potentials for Na^+^ (V_Na_) and K^+^ (V_K_). To determine E_AP_, the energy necessary for generation of action potentials (AP), we calculated the charge (Q) needed to recharge the plasma membrane during an AP. Assuming Q is carried solely by Na^+^ [75], [76], [77], [78], we calculated the amount of ATP expended by the Na^+^/K^+^-ATPase in pumping this amount of Na^+^ back out. Therefore, E_AP_ depends upon AP amplitude (ΔV), specific membrane capacitance (C_s_), surface area of soma (A_soma_) and dendrites (A_dendrite_), and the firing frequency (f). In calculating the energy (E_post_) utilised for postsynaptic excitatory AMPA currents we considered this dependence on f and the flow of charge through glutamate receptors necessary for generating an AP. This charge was estimated from the current threshold for AP generation (I_AP-thr_) and decay time (τ_decay_) of excitatory postsynaptic currents (EPSCs). A detailed description including formulae is given in Text S1. The rationale for disregarding contributions from other neuronal processes is given in the Discussion. All parameters necessary for the calculations were taken from the literature (see Text S1, Fig. S1) and originate from the Mongolian gerbil (unless otherwise noted), the mouse or the rat. Depending on the temperature at which the relevant study was performed, all values were corrected for T = 37°C. All data available for a given parameter were fitted as described in Text S1, and parameters for postnatal ages ranging from 0 to 90 days were calculated utilising the appropriate mathematical function. As the influence of f on calculated energy values is crucial, we give a detailed explanation of our choice of values for f in the Discussion. The values used represent mean firing rates over longer periods of time (>1 s) and we therefore selected as upper limits for f: 100 Hz (before hearing onset), 200 Hz (APs after hearing onset) and 400 Hz (inputs after hearing onset) [18], [48], [75], [77], [79], [80], [81], [82], [83], [84].

## Results

### Developmental Changes in Levels of Metabolic Markers

**Figure 1:**
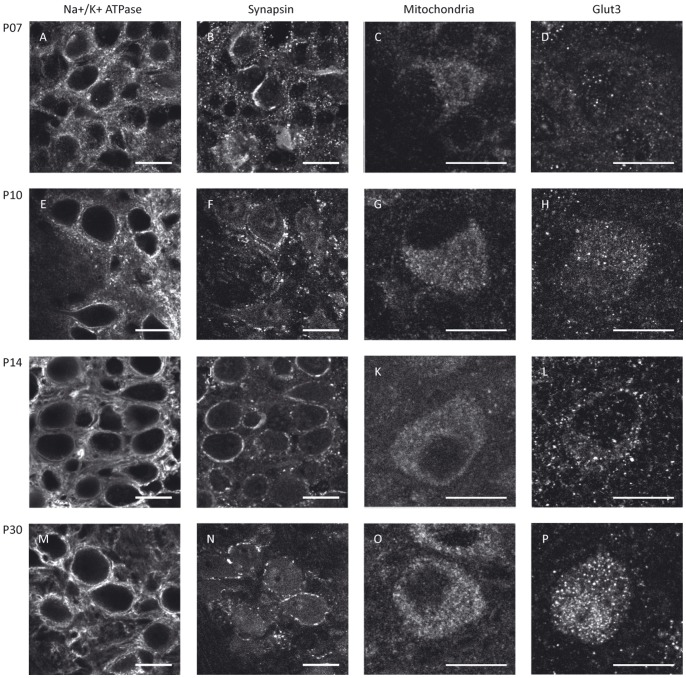
Metabolic maturation in the MNTB. MNTB neurones of Mongolian gerbils at P7 (A–D), P10 (E–H), P14 (I–L) or P30 (M–P) were immunohistochemically stained for Na^+^/K^+^-ATPase (A, E, I, M), synapsin (B, F, J, N), mitochondria (C, G, K, O) or GLUT3 (D, H, L, P). The sample stainings depicted for Na^+^/K^+^-ATPase and synapsin were taken from a double-staining of both markers in the same sections. Scale bar = 20 µm.

**Figure 2:**
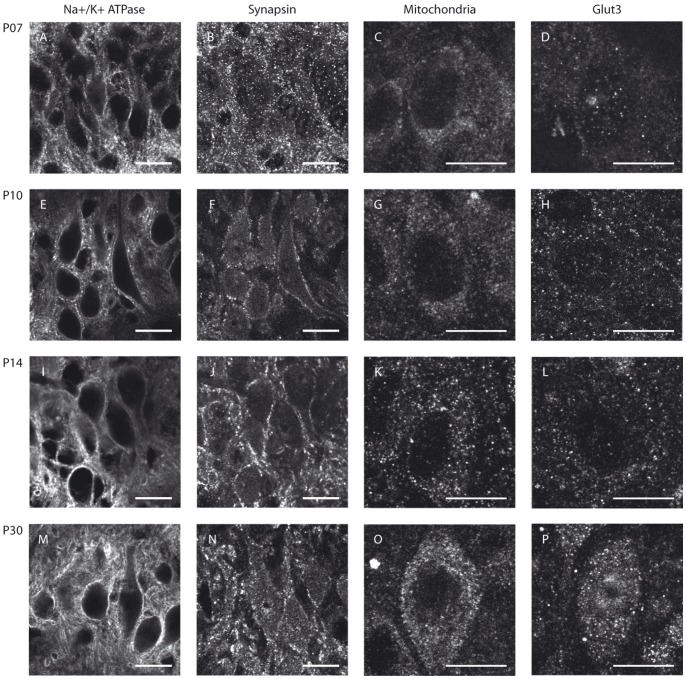
Metabolic maturation in the MSO. MSO neurones of Mongolian gerbils at P7 (A–D), P10 (E–H), P14 (I–L) or P30 (M–P) were immunohistochemically stained for Na^+^/K^+^-ATPase (A, E, I, M), synapsin (B, F, J, N), mitochondria (C, G, K, O) or GLUT3 (D, H, L, P). The sample stainings depicted for Na^+^/K^+^-ATPase and synapsin were taken from a double-staining of both markers in the same sections. Scale bar = 20 µm.

**Figure 3:**
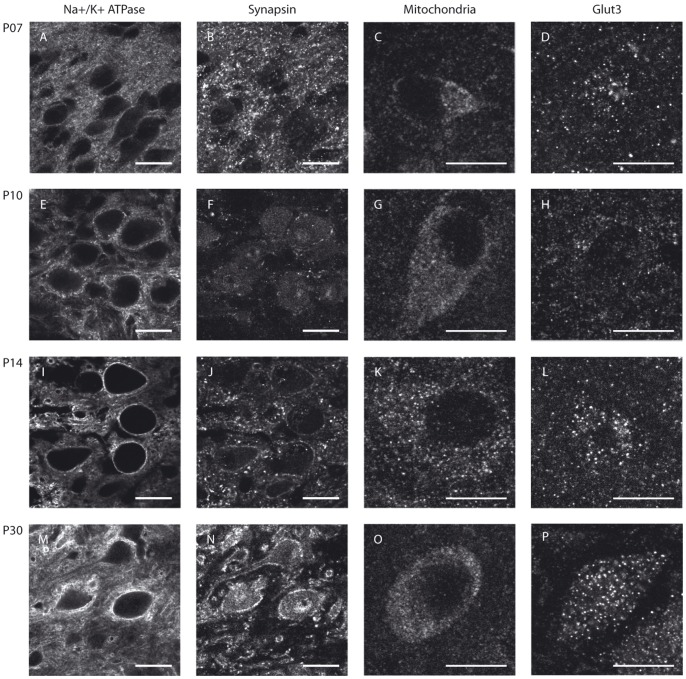
Metabolic maturation in the LSO. LSO neurones of Mongolian gerbils at P7 (A–D), P10 (E–H), P14 (I–L) or P30 (M–P) were immunohistochemically stained for Na^+^/K^+^-ATPase (A, E, I, M), synapsin (B, F, J, N), mitochondria (C, G, K, O) or GLUT3 (D, H, L, P). The sample stainings depicted for Na^+^/K^+^-ATPase and synapsin were taken from a double-staining of both markers in the same sections. Scale bar = 20 µm.

**Figure 4:**
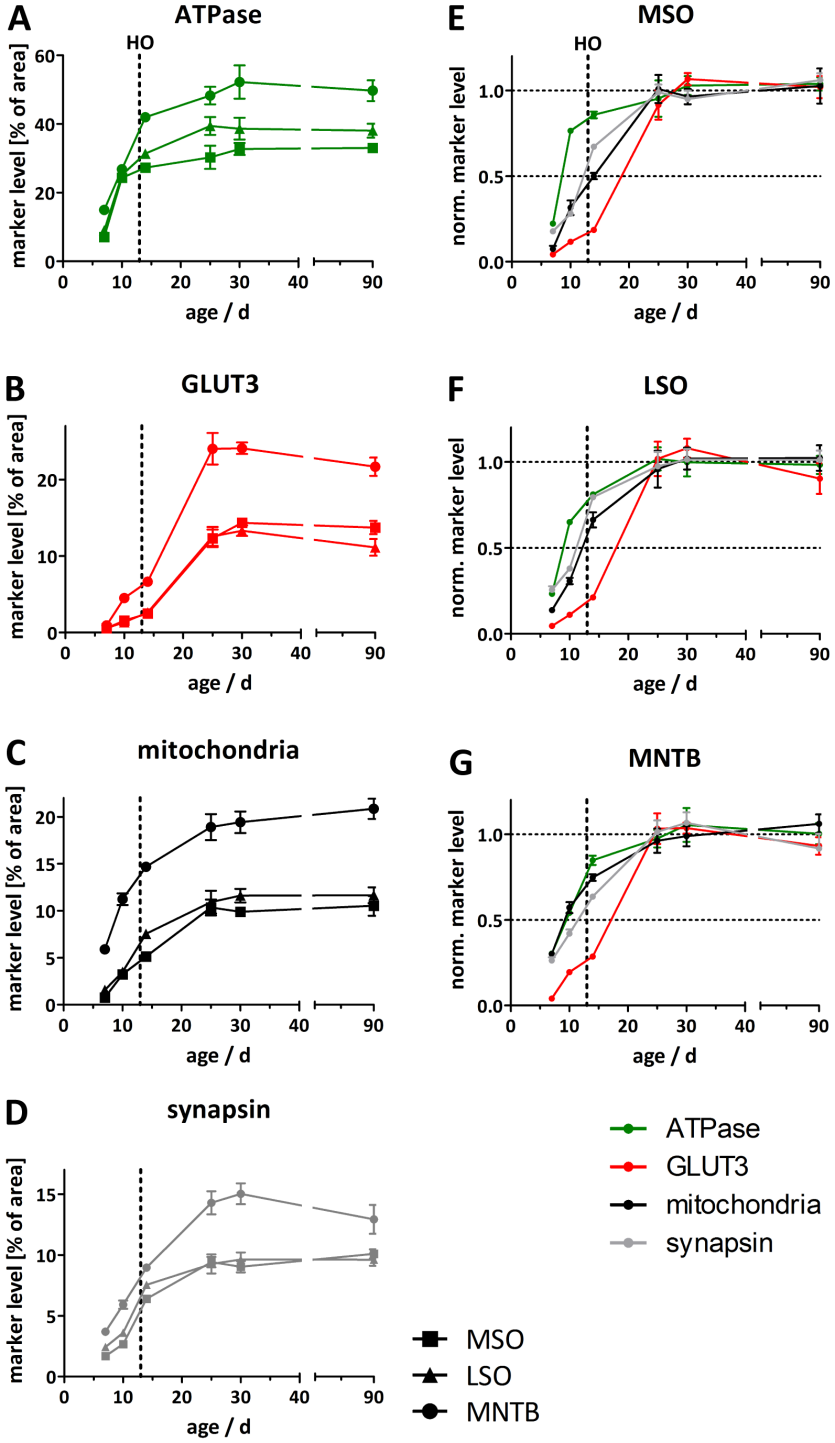
Quantification of relative levels of metabolic markers in the MSO, LSO and MNTB. The marker levels (in % of cross-sectional area) for Na^+^/K^+^-ATPase (A), GLUT3 (B), mitochondria (C) or synapsin (D) are plotted for different ages during the developmental period tested. Circles represent data points obtained from analysing MNTB neurones, squares those from MSO and triangles those from LSO neurones. A comparison of development of metabolic marker levels (Na^+^/K^+^-ATPase, green; GLUT3, red; mitochondria, black; synapsin, grey) in SOC nuclei normalised to mean values of saturation (P25, P30 and P90) is shown for the MSO (E), LSO (F) and MNTB (G). The vertical line represents the time of hearing onset (HO) in the Mongolian gerbil. Data are represented as means ± SEM (n = 5 neurones/data point). Statistical dependence between metabolic marker levels and age was analysed by means of the nonparametric Spearman correlation test. The parameters obtained (Spearman correlation coefficients r; P values) are for the MNTB: ATPase (r = 0.9429; P = 0.0167), GLUT3 (0.8286; 0.0583), mitochondria (1.000; 0.0028), synapsin (0.8286; 0.0583), for the MSO: ATPase (1.000; 0.0028), GLUT3 (0.9429; 0.0167), mitochondria (0.9429; 0.0167), synapsin (0.9429; 0.0167), and for the LSO: ATPase (0.7714; 0.1028), GLUT3 (0.8286; 0.0583), mitochondria (1.000; 0.0028), synapsin (0.9429; 0.0167).

To determine whether metabolic maturation correlates with developmental alterations in the functional activity of SOC neurones, we investigated the density and distribution of several metabolic markers in the nuclei MNTB, MSO and LSO prior to hearing onset (P7 and P10), immediately after hearing onset (P14, “refinement phase”), and at intervals up to the adult stage (P25, P30 and P90) (Figs. 1, 2, 3, 4). The levels of Na^+^/K^+^-ATPase and synapsin as well as mitochondrial density begin to increase before hearing onset in all nuclei investigated (Fig. 4). Na^+^/K^+^-ATPase expression rises early during development with the most prominent up-regulation appearing already between P7 and P10 in the MSO and LSO (Figs. 4A, E and F) and between P7 and P14 in MNTB (Figs. 4A and G). In the MSO, mitochondria and synapsin expression show a more gradual increase over a longer period, between P7 and P25 (Figs. 4C, D and E), whereas their expression patterns in the LSO and MNTB resemble more to that of the Na^+^/K^+^-ATPase. The levels of all studied markers continue to increase until P25, after which their expression levels remain unchanged until P90 (Figs. 4).

**Figure 5:**
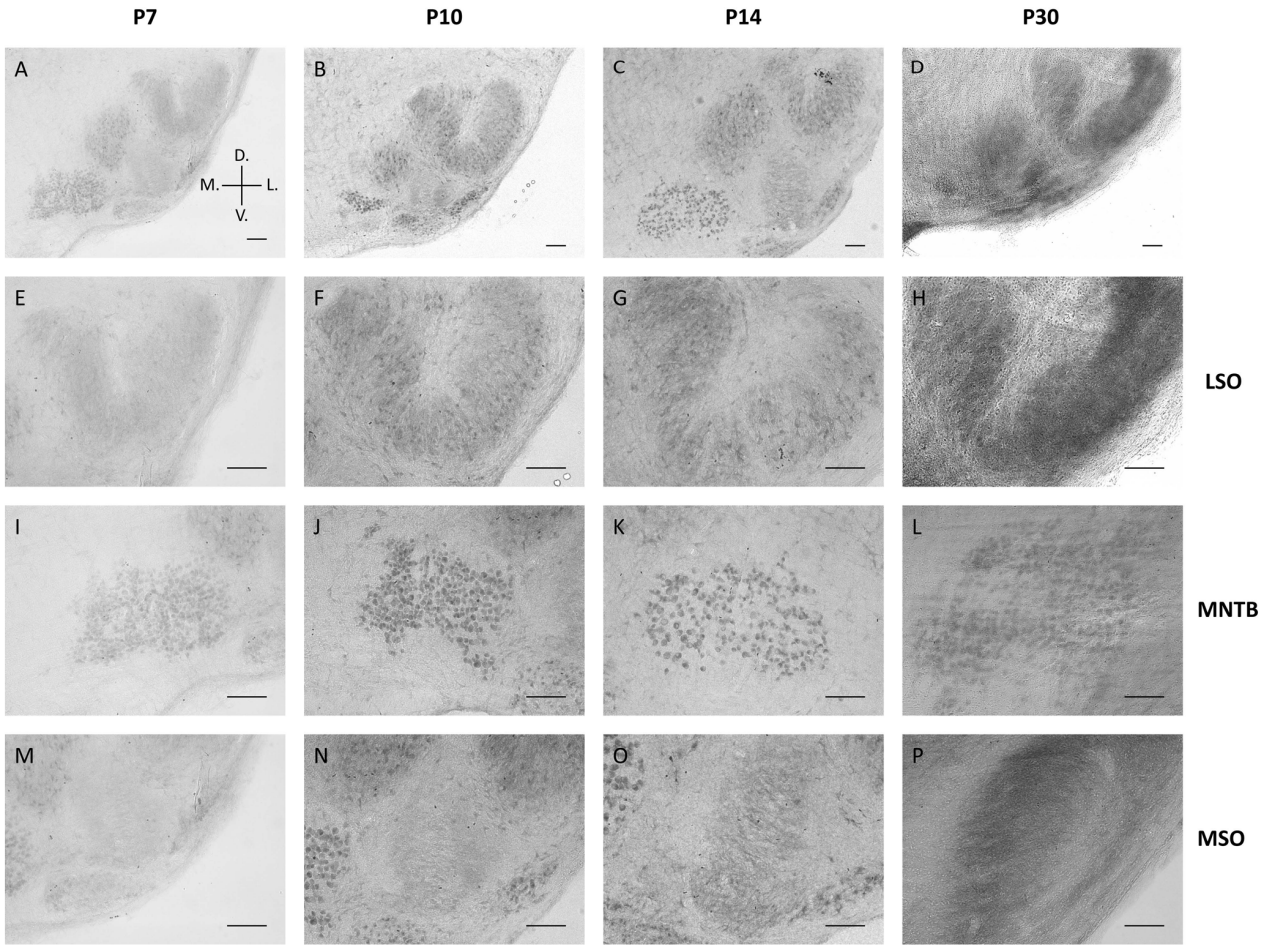
COX activity during development in the auditory brainstem. Representative images of COX activity stainings at different developmental stages of Mongolian gerbils. Images A–D show an overview of the auditory brainstem, E–H show the lateral superior olive (LSO), I–L show the medial nucleus of the trapezoid body (MNTB) and M–P show the medial superior olive (MSO). Images were taken at P7 (A, E, I & M), P10 (B, F, J & N), P14 (C, G, K & O), and P30 (D, H, L & P). Orientation in the brainstem is given in A: D., dorsal; V., ventral; M., medial; L., lateral. Scale bar = 100 µm.

**Figure 6:**
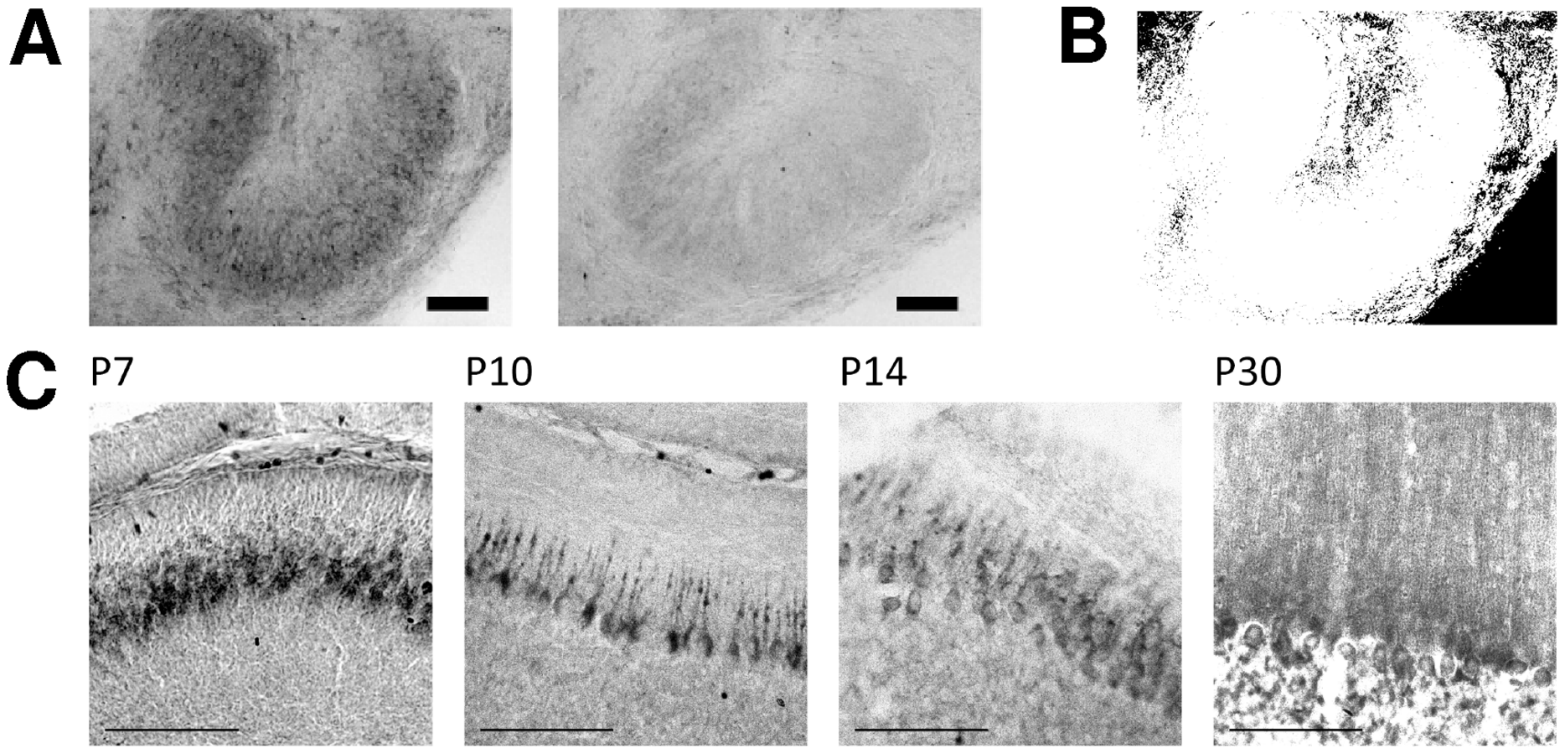
Control experiments for COX activity detection. (A) The majority of the colour reaction is caused by the specific COX activity. The figure depicts one example (LSO, P7) of a control experiment to test for a reaction of unspecific oxidative enzymes. To this, we incubated brain sections in staining solution for COX reaction containing either cytochrome c (left) or no cytochrome c (right). Scale bar = 100 µm. In B the mean average intensity of the unspecific reaction of 3′3-diaminobenzidine was subtracted from the intensity of the specific COX staining. White pixels represent a value above threshold. (C) COX activity in cerebellar Purkinje cells of gerbils aged P7-P30. Scale bars = 100 µm.

The increase in COX activity correlates well with the up-regulation of mitochondria (Figs. 4C and 5). COX activity starts to increase in all investigated nuclei prior to hearing onset, and rises continuously until P30. In order to confirm the specificity of COX activity staining, brain sections were incubated in the normal staining solution containing cytochrome c or in staining solution devoid of cytochrome c, to quantify the background reaction caused by other oxidative enzymes (Fig. 6A). Comparison of the two conditions confirms that the specific COX staining yields a much higher reactivity than the control assay. This is also apparent in the quantification (Fig. 6B): When the average intensity of the non-specific staining is subtracted from the specific COX staining, all pixels lie above the threshold. Only in the area around the nuclei does the intensity appear to be more or less equal under both conditions. In line with this observation, COX activity levels off at P30 (Fig. 5). The intracellular distribution of COX also changes during development. At the early developmental stages investigated, COX is highly abundant in cellular somata, whereas in the adult animals COX activity in the fibres is comparatively increased. This gives rise to a presumably diffuse COX staining at P30, because owing to the similar intensity of staining in somata and surrounding fibres, the different cellular compartments are difficult to discriminate. We compared the results obtained from the auditory brainstem nuclei to the staining of cerebellar Purkinje cells (Fig. 6C) to exclude a general developmental effect, which should be independent of hearing onset. Since the animals begin to move around before the onset of hearing − a task for which the cerebellum is crucial − cerebellar neurones already display a high and behaviourally relevant level of activity at this early stage. No increase of COX activity was observed in cerebellar Purkinje cells during the developmental stages examined, indicating that the changes observed in the auditory brainstem indeed reflect the altered metabolic states of SOC neurones.

Of all the metabolic markers investigated, GLUT3 is the latest to appear. Its levels rise only after the onset of hearing, i.e. between P14 and P25, in all nuclei investigated (Figs. 4B and E–G). In the MSO and LSO, GLUT3 expression is almost undetectable before P14, whereas expression begins earlier in the MNTB, where the transporter is already detectable by P10 (Figs. 1, 2, 3). The anti-GLUT3 antibody is directed against the intracellular C-terminal and the protein exhibits a relatively fast turnover, which accounts for the substantial cytoplasmic staining observed [62], [69].

Among the SOC nuclei investigated, the MNTB stands out. Expression levels of all metabolic markers tested are higher than in the MSO or LSO at all developmental stages examined. The effect is most prominent for GLUT3 expression and mitochondria (Figs. 4B and C). Overall, the time courses of marker expression are similar (Figs. 1, 2, 3, 4). However, COX activity shows its most prominent increase already before hearing onset, i.e. between P7 and P10, and increases only slightly thereafter in the MNTB. In MSO and LSO the increase in COX activity takes place much more gradually between P7 and P30 (Fig. 5).

### Mathematical Modelling of Developmental Changes in Energy Consumption

**Figure 7:**
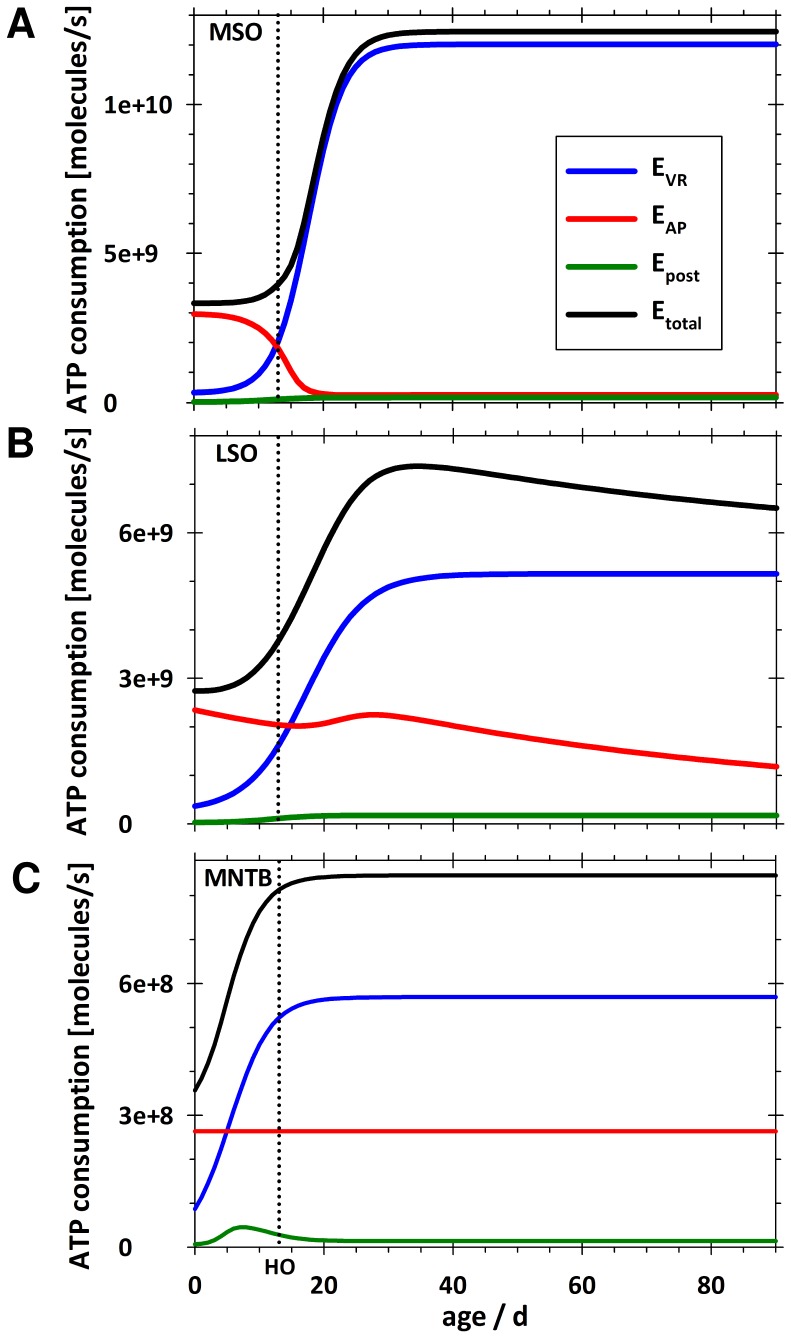
Energy consumption in SOC nuclei (at 100 Hz) increases during maturation. The calculated values for total ATP consumption (E_total_) per cell as well as the individual components E_Vr_ (energy for maintenance of resting membrane potential), E_AP_ (energy for generation of somatic and dendritic APs, and E_post_ (energy for postsynaptic excitatory currents) rise during development for MSO (A), LSO (B), and MNTB (C). The firing frequency for both inputs and postsynaptic AP generation was 100 Hz. Note the difference in total energy for the three nuclei. HO, hearing onset.

A comparison between calculated energy consumption, partitioned between different neuronal processes, in the three SOC nuclei during development is depicted in Fig. 7. A firing frequency of 100 Hz was chosen for all processes, since this value represents the upper limit of spontaneous activity before hearing onset and a reasonable mean activity thereafter (see Discussion). MSO and LSO neurones exhibit similar time courses for E_total_ and for each of the individual components of energy consumption, whereas the profiles for the MNTB show some special features. In both MSO and LSO the E_total_ profile is characterised by a rather shallow rise before hearing onset, which becomes markedly steeper during post-hearing onset refinement phase and levels off around P25 (MSO) and P30 (LSO), respectively. In the LSO a slow decrease over time is observed thereafter. The rise in E_total_ is essentially attributable to an increase in E_Vr_, whilst E_AP_ and E_post_ decrease. This developmental pattern is very pronounced in the MSO, where E_total_ is dominated by E_Vr_ and the two become virtually synonymous after P20. In the MNTB in contrast, E_total_ increases considerably prior to hearing onset and levels off immediately thereafter. Absolute values of E_total_ at saturation are much higher in the MSO than in the other SOC nuclei (2× and 10× higher than in LSO and MNTB, respectively) due to differing relative contributions and absolute values of individual components of energy use.

**Figure 8:**
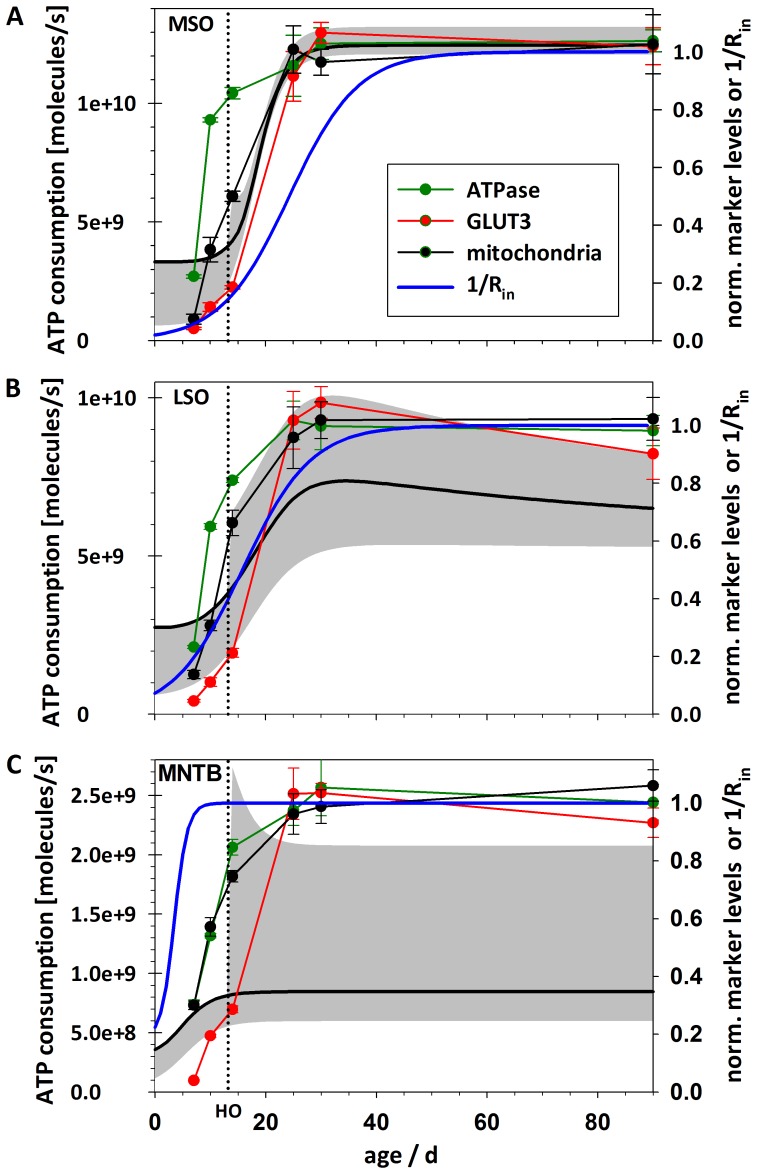
Comparison of time course of total energy consumption (E_total_) and development of metabolic markers. The grey area depicts E_total_ per cell between low (lower margin of area) and high mean firing rates (upper margin of area) for MSO (A), LSO (B), and MNTB (C). For the low rate we used 10 Hz before and after hearing onset (both inputs and postsynaptic AP generation). As an upper estimation we used 100 Hz for all components before hearing onset and after hearing onset 200 Hz (postsynaptic AP generation) and 400 Hz (inputs), respectively. The black line represents the values for 100 Hz before and after hearing onset (both inputs and postsynaptic AP generation) depicted in Fig. 7. HO, hearing onset.

We also compared the developmental trajectory of calculated total ATP consumption (E_total_) with the observed pattern of metabolic marker expression (Fig. 8). As mean firing frequency over longer periods of time are not known, we accounted for this uncertainty by calculating the energy components for a lower (10 Hz) and an upper estimate (100; 200/400 Hz) of mean firing frequency in addition to 100 Hz. Furthermore, Fig. 8 shows the developmental changes in the value of 1/R_in_ which is an important electrophysiological characteristic, especially for leaky adult MSO and LSO neurones, and determines E_Vr_. A detailed description and comparison of the developmental changes depicted in Fig. 8 and their physiological implications follows in the Discussion section.

## Discussion

### Developmental Alterations of Neuronal Energetics

We have studied the maturation of neuronal energy metabolism in brainstem nuclei for the first time by calculating energy consumption using a mathematical model and quantifying metabolic marker levels in three superior olivary complex (SOC) nuclei of the Mongolian gerbil over the first three months of postnatal life (P0–90). In general, the developmental changes in metabolic marker levels parallel the time course of calculated energy consumption in each nucleus. The overall pattern of energy use, and the relative contributions of different neuronal processes to total ATP consumption, varied between the nuclei. Most notably, relative to MSO and LSO, the MNTB exhibits a distinctly earlier and faster maturation of all parameters, which is almost completed by the time of hearing onset. Otherwise, the basic sequence of events is the same in all three nuclei. After the up-regulation of the Na^+^/K^+^-ATPase, the number of mitochondria rises, and this is followed by an increased expression of GLUT3 after the onset of hearing. Na^+^/K^+^-ATPase expression has already reached 85% of its mature level at hearing onset, whereas GLUT3 expression is scarcely detectable prior to that point.

The increase in expression of metabolic markers around the time of hearing onset (P12–13 in gerbils) relates to a process of progressive functional changes in the SOC nuclei, and does not represent a general developmental effect, which coincidentally parallels the maturation of the auditory senses. This finding is supported by a Western blotting study of the rat brainstem, which showed no change in levels of GLUT3 and Na^+^/K^+^-ATPase during the time period P10–14 [78].

One might argue that before hearing onset, neurones could make use of carbon sources other than glucose, such as ketone bodies or lactate [79], [81], [82], [85]. However, glucose is the overwhelmingly predominant fuel for the CNS, whilst other carbon sources are more important during hypoglycaemia [56], and for metabolic cooperation between astrocytes and neurones [79], respectively. Another possibility is that, prior to hearing onset, glucose uptake occurs via a transporter other than GLUT3. However, GLUT3 up-regulation is generally associated with a higher energy demand in neurones [62]. In addition, we found high expression levels already at P10 in MNTB neurones, which is compatible with their generally earlier maturation.

### Physiological Relevance

The expression of the Na^+^/K^+^-ATPase is up-regulated conjointly with mitochondrial density, indicating that metabolic maturation is coupled to the developing capability of these neurones to respond to auditory inputs. This development continues after hearing onset, when relevant auditory inputs arrive in the SOC nuclei and might thus contribute to further maturation, at least in MSO and LSO. The comparatively late up-regulation of GLUT3 relative to the other markers suggests that energy availability is ultimately regulated at the level of glucose uptake into the cell. This makes sense as un-metabolised glucose will affect the composition of the cytoplasm, especially by changing its osmolarity. In addition, high glucose oxidation rates would enhance production of reactive oxygen species and perhaps cause neurotoxicity [83].

Our findings indicate that processing of behaviourally irrelevant spontaneous activity, which occurs before hearing onset and with a rather low firing frequency, is not highly energy consuming. The periods of spontaneous firing are caused by the spontaneous release of ATP by supporting cells in the cochlea [26] and might serve as an important priming cue for the correct development of neuronal circuits and physiological properties of SOC neurones [38], [84], [85]; for review see: [86]. The firing rates range from 0.1 to 110 Hz [13], [87], [88] and we used values of 10 to 100 Hz for our calculations.

The assumed mean firing frequencies are of special importance for the mathematical model, since they affect both E_AP_ and E_post_. In adult animals, when sound-related information from the cochlea arrives in the SOC, maximal mean firing frequencies of 250 Hz for MSO [18], [89] and up to 350 Hz for LSO [48], [77] and MNTB [75], [77], [90], [91], [92] have been measured. In the refinement phase, an age-related rise in maximal frequency was observed as well [18], [48], [93], [94]. Utilising the mathematical model we calculated mean energy consumption over long periods of time (>1s). As maximal firing frequencies given in the literature are normally maintained for only several 100 ms, they most probably overestimate the true physiological values. We have therefore worked with f values ranging from 10 Hz to 200 Hz postsynaptic AP generation, and 400 Hz for presynaptic inputs. This range, together with that for pre-hearing onset neurones, gives a reasonable estimate of the range of ATP consumption rates in the neurones studied.

Glucose uptake as well as ATP production and consumption are assumed to proceed at rather low level under resting conditions, but the neurone must be capable of responding to high energy demands during periods of high neuronal activity. Hence, the necessary numbers of mitochondria, enzymes and transporters must be available and potentially functional. And indeed, that is what our immunohistochemical studies revealed. Therefore, it seems most appropriate to compare the marker levels with calculated energy values for the highest frequencies used. For low-frequency firing (10 Hz), the total ATP consumption (E_total_) is mainly dominated by energy used to maintain the resting membrane potential. As E_Vr_ values are in turn mainly determined by R_in_, the time course of E_total_ parallels that of 1/R_in_. The provision of a wide dynamic range of firing frequencies between 0 and almost 1000 Hz seems to depend on a dynamic metabolic range. Particularly in the MNTB, the difference in total ATP consumption associated with different firing frequencies is enormous.

### MSO & LSO

Whilst the temporal profile of metabolic marker levels and calculated energy values are very consistent for all three nuclei, the absolute values differ considerably. MNTB marker levels are clearly higher than for MSO/LSO, whereas E_total_ declines in the order MSO>LSO>MNTB. How can the high calculated values in the MSO be explained? Quite obviously, E_total_ is determined by E_Vr_, and thus, by R_in_ irrespective of firing frequency. Low R_in_ values due to a high membrane conductance in MSO neurones are a prerequisite for their main function, i.e. highly precise coincidence detection in the µs range. Therefore, we conclude that in case of the MSO, temporally accurate input integration needs more energy than the attainment of high activity levels. This implies that metabolic demands rise with the development of the ability to localise sources of sound.

### MNTB

The metabolic maturation of the MNTB deviates from that of the MSO and LSO in a number of respects, and this is reflected in the results of both mathematical modelling and the immunohistochemical study of metabolic markers. Essentially in the MNTB, changes begin earlier and proceed at a faster pace. For example, mitochondrial density in the MNTB has reached half-saturation values by P10− a level not attained in MSO/LSO before hearing onset. GLUT3 expression becomes evident by P10, whereas in the other nuclei GLUT3 is almost un-detectable before hearing onset, in agreement with a previous report [60]. Since GLUT3 is responsible for most glucose uptake, this observation supports the argument that MNTB neurones mature early. Our results are in agreement with data from other groups, who have reported early structural and functional maturation of the MNTB by P2–P5 compared to other SOC nuclei [27], [84], [87] and shown that electrophysiological features of principal MNTB neurones remain constant after P14 [40]. Accordingly, the large calyces of Held are already clearly visible by P7 in sections stained for synapsin. Absolute levels of all markers are clearly higher in MNTB than in MSO and LSO, and by P10, they have almost reached values that are seen in MSO and LSO neurones only at saturation, which has been reported for mitochondrial density, COX activity and GLUT3 levels in adult animals [15], [16], [88].

Why are the absolute levels of metabolic markers at their highest in the MNTB, while the calculated values for ATP consumption are the lowest? One explanation would be that spontaneous activity occurs in the MNTB after hearing onset [13] and exhibits an even higher firing frequency than before [73]. Another reason could be the high frequency firing bursts (up to 800 Hz) of very short duration (a few ms) observed in the developing MNTB [89]. Both would make higher frequency firing more likely for the MNTB than for the other nuclei. This was not considered in our calculation, but it would increase E_AP_ and E_post_ and hence E_total_ for the MNTB and could raise energy values to levels close to those of MSO and LSO. The MNTB with its calyx of Held synapse could also be energetically costly for other reasons. We assumed equal efficiency factors (EF) for AP generation in all nuclei, but the EF in the MNTB might well be higher than in other nuclei and, as a consequence, ATP consumption in the MNTB could be higher than calculated. Finally, there are the energy-consuming processes, which were not considered in our mathematical model, but would be reflected in metabolic marker levels. Due to the specialisation of the large MNTB synapse, larger numbers of ion channels and transporters might be expressed in the postsynaptic membrane. Expression and turnover of these proteins, as well as postsynaptic neurotransmitter uptake and metabolism consume energy and, therefore, housekeeping processes might require more energy in the MNTB than in other nuclei. All these factors - individually or in combination - could result in the true energy consumption being higher than calculated by our model.

### Mathematical Model

Our use of the mathematical model developed by Attwell and others [4], [5], [6], [50] to illuminate the neuroenergetics of the SOC nuclei proves that it can be successfully adapted to specialised auditory neurones on the one hand and developmental issues on the other. A comparison with results on cerebral cortex, cerebellum and olfactory glomerulus shows a similar range of absolute energy consumption, and of variability in the fractional contribution of different neuronal processes [4], [5], [6], [50]. Like every other model, it is based on several assumptions, which have been debated in the literature [5], [6], [50] and are discussed in detail below.

The energy-consuming tasks we considered are: maintenance of resting membrane potential, generation of action potentials and postsynaptic excitatory currents, as these have been shown to be the most prominent sources of energy expenditure in other neurones, and are closely related to the postsynaptic localisation of the metabolic markers we have quantified. With regard to AP generation we included somatic and dendritic components, but excluded axonal excitability. As the axonal constituent is sufficiently provided for by local ATP production [90], it is not related to the metabolic marker localisation we analysed. Postsynaptic inhibitory currents were disregarded because the metabolic demand for inhibitory neurotransmission is assumed to be much lower than for the excitatory signalling [91], [92], [93]. It is estimated that regeneration of the Cl^−^ gradient requires less than 1% of the energy needed to implement an equivalent change in the Na^+^ gradient [5], [6], [50], since Cl^−^ has to be transported against a shallower electrochemical gradient. Therefore developmental changes in intracellular Cl^−^ concentrations [94], [95], [96], [97] are irrelevant (see below). Postsynaptic metabotropic processes are assumed to be energetically less demanding [5], [6], [50]. NMDA currents in the MNTB are treated as AMPA currents in the estimates outlined in Text S1. Neurotransmitter recycling was not included in our calculations, since various cell types (e.g. glial cells) are involved and the contribution of the postsynaptic neurone has been calculated to be rather small [5], [6], [50]. The same holds for Ca^2+^-dependent, presynaptic processes involved in neurotransmitter release [5], [6], [50], which are, moreover, not related to the postsynaptic localisation of our metabolic markers. The amount of energy a neurone expends on “housekeeping” functions unrelated to neuronal excitability is not known, although some authors assume it to be in the range of 25% of the cellular energy consumption. In light of this uncertainty, we have not considered this parameter, but will discuss its implications below.

### Estimation of Model Parameters

The electrophysiological and morphological parameters plugged into the mathematical model were extracted from the literature. Wherever possible, the values derive from studies on the gerbil, but they were also taken from mouse and rat, whose developmental programmes and time point of hearing onset are comparable to the gerbil’s [26], [74], [84], [98], [99], [100]. An exception must be made for the MSO, which is related to low-frequency hearing, and among rodents, is only substantially present in the gerbil. Since the datasets available for different parameters differ in size, their accuracy varies. In addition, the age range of published data determines the accuracy of the parameters extrapolated to a range P0–90. In cases, where data for a certain parameter were only available for one age group, uniformity was assumed or, if physiologically reasonable, an age dependency similar to that in one of the other SOC nuclei was employed. The latter approach was also used if no data at all were available for a particular parameter in a given nucleus. A detailed description of parameter estimation, including references, can be found in Text S1. In all nuclei, R_in_, I_AP-thr_ and τ_decay_ change with maturation, with R_in_ having the largest published dataset. In some of the nuclei, the following parameters also undergo maturation: ΔV_soma_ (MSO), ΔV_dendrite_ (MSO), A_dendrite_ (MSO, LSO), whilst V_r_, and A_soma_ are constant in all of them. We chose an AP efficiency factor of 2, which represents the upper limit of the latest published data (for discussion see [2]) and is close to the value of 4, which was used for similar calculations and is based on results of Hodgkin [101]. Concentrations for Na^+^ and K^+^, and hence the corresponding reversal potentials, were chosen to be constant due to lack of data. However, as developmental alteration of intracellular Cl^−^ concentrations has been reported in the SOC [94], [95], [96], [97], a dependence of Na^+^ and K^+^ concentrations on age cannot be excluded. A possible contribution to V_r_ and R_in_ from a potential Cl^−^ conductance was neglected due to missing data as well.

### Conclusions

Using a combination of quantitative immunohistochemistry and mathematical modelling of energy consumption, we were able to describe generalised developmental schemes as well as nucleus-specific variations for the major components of the SOC in the gerbil. We found that the period preceding hearing onset is most crucial for the MNTB, whilst in MSO/LSO the refinement phase after hearing onset represents a pivotal developmental phase. Neuronal input resistance (R_in_), which represents an important electrophysiological parameter and can be most reliably obtained from the literature for a wide age range, dominates energy consumption, especially in leaky MSO neurones and at low firing frequencies. For the MSO, we propose that coincidence detection with its requirement for temporal precision and high-frequency phase-locking represents a metabolically more costly process than high-frequency firing itself. For the MNTB, we suggest that cellular processes related to its specialised type of synapse contribute to ATP consumption and account for the observed discrepancies between immunohistochemical quantification and mathematical modelling. Further detailed experimental and modelling efforts are necessary, if we are to understand neuroenergetics in the SOC. A comparative analysis of further auditory neurones, as well as functional studies (e.g. NAD(P)H and flavoprotein imaging) in acute brain slices in combination with electrophysiological recordings could deepen our understanding of neuroenergetics. It has long been known that neurones produce only as much energy as they require [51] and our data support this assumption. For other sensory systems such as the visual system of the macaque, it has been demonstrated that inactivation of the sensory pathway decreases the activity of COX [102], [103]. A reduction of COX activity in auditory brainstem nuclei has been reported either caused by conductive hearing loss in Mongolian gerbils [104] or following unilateral deafening of cats [105]. It would, thus, be intriguing to investigate whether deafening of gerbils before the onset of hearing interferes with the observed maturation of metabolic markers in auditory brainstem nuclei.

## Supporting Information

Figure S1 **Dependence of electrophysiological parameters on age.** Absolute (top) and relative values (bottom) for all three SOC nuclei.
(TIF)

Text S1 **Description of the mathematical model.**
(DOC)
