# Supplementary figures and images for: Metabolic Maturation of Auditory Neurones in the Superior Olivary Complex
Source: PLoS One. 2013 Jun 27;8(6):e67351. doi: 10.1371/journal.pone.0067351 (PMC3694961; doi:10.1371/journal.pone.0067351)

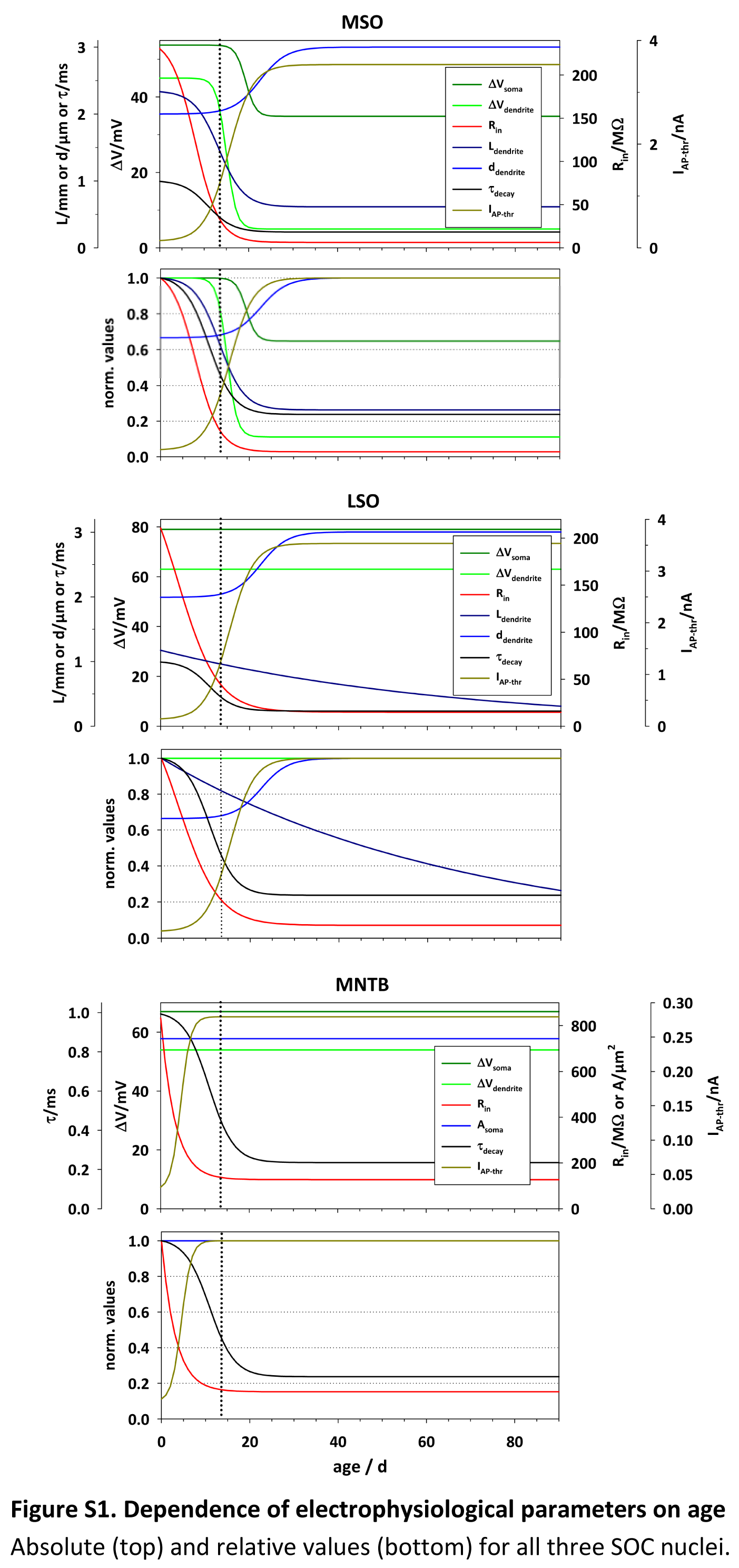

Supplement: Figure S1 — Dependence of electrophysiological parameters on age. Absolute (top) and relative values (bottom) for all three SOC nuclei. (TIF) [file pone.0067351.s001.tif]
