# Supplementary material for: Metabolic Maturation of Auditory Neurones in the Superior Olivary Complex
Source: PLoS One. 2013 Jun 27;8(6):e67351. doi: 10.1371/journal.pone.0067351 (PMC3694961; doi:10.1371/journal.pone.0067351)
Supplement: Text S1 — Description of the mathematical model. (DOC) [file pone.0067351.s002.doc]

**Text S1: Model description**

Parameters were derived from published data on the Mongolian gerbil (unless otherwise noted), the mouse or the rat. In fitting the dependence of a certain parameter on age, weighting for the number of cells per age group was applied. Hence, either published mean values with errors were taken or individual data points were extracted from published scatter plots using the software DigitizeIt 1.5 (http://www.digitizeit.de).

The rate of ATP consumption required for maintenance of the resting membrane potential (E_Vr_) was calculated in molecules per second per cell using formula {1}

E_Vr_ = I_Na_/(3F) = N_L_*(V_Na_ - V_r_)*(V_r_ - V_K_)/[F*R_in_*(V_r_ + 2V_Na_ - 3V_K_)] {1}

where F = 96485 C/mol; N_L_ = 6.022*10^23^ mol^-1^; V_Na_ = Na^+^ reversal potential = 50 mV; V_K_ = K^+^ reversal potential = -100 mV. For V_Na_ and V_K_ typical values were chosen and assumed to be constant according to the literature .

The rate of ATP consumption for AP generation (E_AP_) was calculated (in molecules per second per cell) using formula {2}

E_AP_ = f*EF*n(Na^+^)/3 = f*EF*Q/(3e) = f*EF*C*ΔV/(3e) = f*EF*A*C_s_*ΔV/(3e) {2}

where f = firing frequency; EF = efficiency factor; e = elementary electrical charge = 1.6*10^-19^ C; ΔV = AP amplitude (relative to V_r_); C_s_ = specific membrane capacity = 1 µF/cm^2^; A = surface area of the cell compartment. In many studies, a value of 4 was chosen for EF, based on the results of Hodgkin . In more recent publications a factor between 1 and 2 was chosen, based on recent experimental results (for a discussion and values in the cerebral cortex and the cerebellum see ). In this study we used a value of 2. The soma was assumed to be a spheroid and its surface area was calculated with formula {3}


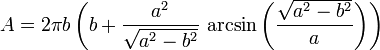
 {3}

where a = the longer semi-axis, b = the shorter semi-axis. The dendrites were assumed to be circular cylinders and their total surface area was calculated as π*d*L, where d = mean diameter and L = total length of dendrites. Values for somatic APs were chosen only from studies that had been carried out at physiological temperatures, since low-temperature recordings produce non-physiologically high AP amplitudes. Estimation of dendritic APs is difficult, as it is highly dependent on neurone type and has rarely been studied (for estimations see below).

The rate of ATP consumption accompanying postsynaptic excitatory currents (E_post_) was calculated using formula {4}

E_post_ = f*I_AP-thr_*τ_decay_ / (3e) {4}

where f = firing frequency; I_AP-thr_ = current threshold for AP generation; τ_decay_ = time constant of EPSC decay; e = elementary electrical charge = 1.6*10^-19^ C. Postsynaptic excitatory currents were assumed to be carried by Na^+^ and K^+^. As in the calculation of ATP consumption by the Na^+^/K^+^-ATPase, only Na^+^ flows were considered. We calculated the pure Na^+^ current (I_Na_(V_h_)) at the holding potential (V_h_) from the published current I_AP-thr_(V_h_) using formula {5}

I_Na_(V_h_) = I_AP-thr_(V_h_)*(V_h_-V_Na_)/(1.5V_h_-V_Na_-0.5V_K_) {5}

where V_Na_ = Na^+^ reversal potential = 50 mV; V_K_ = K^+^ reversal potential = -100 mV. The value at V_r_ was then calculated with formula {6}

I_AP-thr_(V_r_) = I_Na_(V_h_)*V_r_/V_h_. {6}

The values for I_AP-thr_(V_r_) and τ_decay_ were corrected for deviations from 37°C utilising Q_10_ values of 1.4 for current amplitudes and 1.7 for decay times, respectively.

***Parameters for MSO neurones***

Fig. S1 depicts all parameters as absolute and relative values. A constant value of V_r_ = -64 mV was chosen . Data for R_in_ were taken from several publications and fitted by a sigmoidal function with variable slope {7}

y = bottom + (top-bottom)/(1+10^((LogEC_50_-X)*Hillslope)) {7}

yielding the following parameters: bottom = 6.342; top = 242.4; LogEC_50_ = 7.909; Hillslope = -0.1607. The surface area of the soma was calculated to equal 937 µm^2^ and regarded as constant . Fitting of somatic AP amplitude data from different published studies on gerbils with a Boltzmann sigmoidal function {8}

y = bottom + (top-bottom)/(1+exp((V_50_-X)/slope)) {8}

results in the following parameters: bottom = 53.78; top = 34.88; V_50_ = 19.28; slope = 1.115. It is known that dendritic APs disappear after hearing onset to preclude AP back-propagation . Therefore, we used the same Boltzmann function {8} as for somatic APs with equal slope, yielding: bottom = 45; top = 5; V_50_ = 15; slope = 1.115. The total length of dendrites was calculated based on fitting of morphological data from the gerbil with a Boltzmann function {8}, yielding: bottom = 2.348; top = 0.6135; V_50_ = 13.28; slope = 2.791. The same procedure was applied to data on dendrite diameter (d) from the same publication and a minimal d of 2 µm at P0 and a maximum of 3 µm at P90 was assumed, which gave the following parameters: bottom = 2.0; top = 3.0; V_50_ = 22.5; slope = 3.0. Data for I_AP-thr_ in the gerbil were fitted with a Boltzmann sigmoidal function {8}, and the following fit parameters were obtained: bottom = 0.1307; top = 3.534; V_50_ = 15.44; slope = 2.695. For EPSC decay, values from the MNTB were used (see below) since they agree with values in adult gerbils .

***Parameters for LSO neurones***

A constant V_r_ of -63 mV was chosen, as published for the gerbil . Values for R_in_ were taken from gerbil, mouse and rat and can be fitted with a sigmoidal function {7} with the following parameters: bottom = 15.02; top = 293.5; LogEC_50_ = 3.872; Hillslope = -0.09659. For somatic AP amplitude a constant value of 79 mV was assumed, based on data from rat and mouse . A constant somatic surface area of 1256 µm^2^ was calculated using data from the rat and assuming a spherical shape . Dendritic APs are assumed to be constant and 20% smaller than somatic APs. In contrast to the MSO, they are not assumed to disappear, since their presence is a crucial prerequisite for AP back-propagation . For total dendritic length an exponential decay was assumed based on gerbil data , which gave the following parameters: y_0_ = 1.1750; plateau = 0; k = 0.015. Due to the lack of data, MSO values for dendritic diameter were used (see above). As no LSO data on I_AP-thr_ are available in the literature, we calculated the value according to the Boltzmann fit parameters for the MSO (see above). For EPSC decay, MNTB values have been used (see below).

***Parameters for MNTB neurones***

A constant V_r_ of -67 mV was used based on data in rat and mouse . Changes in R_in_ can be described by an exponential decay based on data from gerbil and rat . The fit parameters are: y_0_ = 833.7; plateau = 127.2; k = 0.3170. A constant mean value of 743 µm^2^ was used for somatic surface area, based on studies in mouse and rat . A constant value of 67 mV for somatic AP amplitude was calculated from studies carried out at near physiological temperature in gerbil, mouse and rat . The total dendritic surface area was assumed to be 25% of that of the soma , i.e. equal to 248 µm^2^. Dendritic APs are assumed to be constant and 20% smaller than somatic ones. Data on I_AP-thr_ in the gerbil , rat and mouse were fitted with the Boltzmann sigmoidal function {8}. The following fit parameters were obtained: bottom = 0.0272; top = 0.2796; V_50_ = 4.524; slope = 1.112. In contrast to the other nuclei, NMDA receptors contribute significantly to the EPSC in the MNTB . However, the level of ATP consumption attributable to EPSCs carried by NMDA receptors is only slightly higher than those carried by AMPA receptors, since the transport of one Ca^2+^ ion by the NMDA receptors uses one ATP molecule. The same holds for Ca^2+^-permeable AMPA receptors. For EPSC decay, abundant values from mouse and rat were used , and fitted by a sigmoidal fit function {7} yielding the following parameters, which were also used for MSO and LSO: bottom = 0.2356; top = 1.0080; LogEC_50_ = 10.9100; Hillslope = -0.1558.

**References**

1. Attwell D, Laughlin SB (2001) An energy budget for signaling in the grey matter of the brain. Journal of cerebral blood flow and metabolism : official journal of the International Society of Cerebral Blood Flow and Metabolism 21: 1133-1145.

2. Howarth C, Peppiatt-Wildman CM, Attwell D (2010) The energy use associated with neural computation in the cerebellum. Journal of cerebral blood flow and metabolism : official journal of the International Society of Cerebral Blood Flow and Metabolism 30: 403-414.

3. Hodgkin A (1975) The optimum density of sodium channels in an unmyelinated nerve. Philosophical transactions of the Royal Society of London Series B, Biological sciences 270: 297-300.

4. Howarth C, Gleeson P, Attwell D (2012) Updated energy budgets for neural computation in the neocortex and cerebellum. Journal of cerebral blood flow and metabolism : official journal of the International Society of Cerebral Blood Flow and Metabolism 32: 1222-1232.

5. Chirila FV, Rowland KC, Thompson JM, Spirou GA (2007) Development of gerbil medial superior olive: integration of temporally delayed excitation and inhibition at physiological temperature. J Physiol 584: 167-190.

6. Couchman K, Grothe B, Felmy F (2010) Medial superior olivary neurons receive surprisingly few excitatory and inhibitory inputs with balanced strength and short-term dynamics. The Journal of neuroscience : the official journal of the Society for Neuroscience 30: 17111-17121.

7. Magnusson AK, Kapfer C, Grothe B, Koch U (2005) Maturation of glycinergic inhibition in the gerbil medial superior olive after hearing onset. J Physiol 568: 497-512.

8. Scott LL, Mathews PJ, Golding NL (2005) Posthearing developmental refinement of temporal processing in principal neurons of the medial superior olive. J Neurosci 25: 7887-7895.

9. Rautenberg PL, Grothe B, Felmy F (2009) Quantification of the three-dimensional morphology of coincidence detector neurons in the medial superior olive of gerbils during late postnatal development. J Comp Neurol 517: 385-396.

10. Scott LL, Hage TA, Golding NL (2007) Weak action potential backpropagation is associated with high-frequency axonal firing capability in principal neurons of the gerbil medial superior olive. The Journal of physiology 583: 647-661.

11. Walcher J, Hassfurth B, Grothe B, Koch U (2011) Comparative posthearing development of inhibitory inputs to the lateral superior olive in gerbils and mice. J Neurophysiol 106: 1443-1453.

12. Kandler K, Friauf E (1995) Development of electrical membrane properties and discharge characteristics of superior olivary complex neurons in fetal and postnatal rats. Eur J Neurosci 7: 1773-1790.

13. Wu SH, Kelly JB (1991) Physiological properties of neurons in the mouse superior olive: membrane characteristics and postsynaptic responses studied in vitro. Journal of neurophysiology 65: 230-246.

14. Rietzel HJ, Friauf E (1998) Neuron types in the rat lateral superior olive and developmental changes in the complexity of their dendritic arbors. The Journal of comparative neurology 390: 20-40.

15. Sanes DH, Song J, Tyson J (1992) Refinement of dendritic arbors along the tonotopic axis of the gerbil lateral superior olive. Brain Res Dev Brain Res 67: 47-55.

16. Forsythe ID, Barnes-Davies M (1993) The binaural auditory pathway: excitatory amino acid receptors mediate dual timecourse excitatory postsynaptic currents in the rat medial nucleus of the trapezoid body. Proc Biol Sci 251: 151-157.

17. Borst JG, Helmchen F, Sakmann B (1995) Pre- and postsynaptic whole-cell recordings in the medial nucleus of the trapezoid body of the rat. The Journal of physiology 489 ( Pt 3): 825-840.

18. Rusu SI, Borst JG (2011) Developmental changes in intrinsic excitability of principal neurons in the rat medial nucleus of the trapezoid body. Developmental neurobiology 71: 284-295.

19. Inchauspe CG, Forsythe ID, Uchitel OD (2007) Changes in synaptic transmission properties due to the expression of N-type calcium channels at the calyx of Held synapse of mice lacking P/Q-type calcium channels. The Journal of physiology 584: 835-851.

20. Lorteije JA, Rusu SI, Kushmerick C, Borst JG (2009) Reliability and precision of the mouse calyx of Held synapse. The Journal of neuroscience : the official journal of the Society for Neuroscience 29: 13770-13784.

21. Hoffpauir BK, Kolson DR, Mathers PH, Spirou GA (2010) Maturation of synaptic partners: functional phenotype and synaptic organization tuned in synchrony. The Journal of physiology 588: 4365-4385.

22. Berntson AK, Walmsley B (2008) Characterization of a potassium-based leak conductance in the medial nucleus of the trapezoid body. Hearing research 244: 98-106.

23. Hoffpauir BK, Grimes JL, Mathers PH, Spirou GA (2006) Synaptogenesis of the calyx of Held: rapid onset of function and one-to-one morphological innervation. J Neurosci 26: 5511-5523.

24. Taschenberger H, Leao RM, Rowland KC, Spirou GA, von Gersdorff H (2002) Optimizing synaptic architecture and efficiency for high-frequency transmission. Neuron 36: 1127-1143.

25. Leao RN, Leao RM, da Costa LF, Rock Levinson S, Walmsley B (2008) A novel role for MNTB neuron dendrites in regulating action potential amplitude and cell excitability during repetitive firing. The European journal of neuroscience 27: 3095-3108.

26. Tong H, Steinert JR, Robinson SW, Chernova T, Read DJ, et al. (2010) Regulation of Kv channel expression and neuronal excitability in rat medial nucleus of the trapezoid body maintained in organotypic culture. The Journal of physiology 588: 1451-1468.

27. Hardman RM, Forsythe ID (2009) Ether-a-go-go-related gene K+ channels contribute to threshold excitability of mouse auditory brainstem neurons. The Journal of physiology 587: 2487-2497.

28. Case DT, Gillespie DC (2011) Pre- and postsynaptic properties of glutamatergic transmission in the immature inhibitory MNTB-LSO pathway. Journal of neurophysiology 106: 2570-2579.

29. Youssoufian M, Oleskevich S, Walmsley B (2005) Development of a robust central auditory synapse in congenital deafness. Journal of neurophysiology 94: 3168-3180.

30. Chuhma N, Koyano K, Ohmori H (2001) Synchronisation of neurotransmitter release during postnatal development in a calyceal presynaptic terminal of rat. The Journal of physiology 530: 93-104.

31. Chuhma N, Ohmori H (1998) Postnatal development of phase-locked high-fidelity synaptic transmission in the medial nucleus of the trapezoid body of the rat. The Journal of neuroscience : the official journal of the Society for Neuroscience 18: 512-520.

32. Joshi I, Shokralla S, Titis P, Wang LY (2004) The role of AMPA receptor gating in the development of high-fidelity neurotransmission at the calyx of Held synapse. The Journal of neuroscience : the official journal of the Society for Neuroscience 24: 183-196.

33. Joshi I, Wang LY (2002) Developmental profiles of glutamate receptors and synaptic transmission at a single synapse in the mouse auditory brainstem. The Journal of physiology 540: 861-873.

34. Taschenberger H, von Gersdorff H (2000) Fine-tuning an auditory synapse for speed and fidelity: developmental changes in presynaptic waveform, EPSC kinetics, and synaptic plasticity. The Journal of neuroscience : the official journal of the Society for Neuroscience 20: 9162-9173.

35. Muller J, Reyes-Haro D, Pivneva T, Nolte C, Schaette R, et al. (2009) The principal neurons of the medial nucleus of the trapezoid body and NG2(+) glial cells receive coordinated excitatory synaptic input. The Journal of general physiology 134: 115-127.
